# Use of physical activity by occupational therapists and speech-language therapists in KwaZulu-Natal

**DOI:** 10.4102/safp.v67i1.6184

**Published:** 2025-09-30

**Authors:** Onika Makaula, Ntandoyenkosi L. Msomi, Andrew J. Ross

**Affiliations:** 1 Department of Public Health Medicine, College of Health Sciences, University of KwaZulu-Natal, Durban, South Africa; 2 Department of Family Medicine, College of Health Sciences, University of KwaZulu-Natal, Durban, South Africa

**Keywords:** physical activity, rehabilitation, occupational therapy, speech and language therapy, ICF, qualitative

## Abstract

**Background:**

Physical activity (PA) plays an important role in rehabilitation by promoting emotional well-being, functional independence, and both physical and cognitive recovery. Although its application in rehabilitation varies, both occupational therapists and speech and language therapists incorporate PA to address individualised patient needs.

**Objective:**

We explored the use of PA by occupational therapists and speech and language therapists in the integration of PA in treatment.

**Methods:**

Ten therapists participated in virtual semi-structured interviews to explore their use of PA in patient treatment. A directed content analysis guided by the International Classification of Functioning, Disability and Health (ICF) framework was employed. NVivo software facilitated the coding of data into pre-established ICF categories: body structures and functions, activities and participation, and environmental factors.

**Results:**

Therapists used PA to achieve endurance, strength and cognitive recovery (body structures and functions); promote engagement in meaningful tasks (activities and participation); and identify environmental barriers and facilitators, such as resource constraints and interdisciplinary collaboration (environmental factors).

**Conclusion:**

PA is regarded as a valuable therapeutic tool across ICF domains, with its optimal use in public sector facilities being hindered by institutional challenges, including staff shortages and limited resources.

**Contribution:**

This study contributes to the understanding of how occupational therapists and speech and language therapists integrate PA into rehabilitation, capturing practical clinical strategies aligned with the ICF framework.

## Background

Integrating physical activity (PA) in rehabilitation services remains an important yet underexplored area globally, particularly within occupational therapy and speech-language therapy. According to Dhuli and colleagues,^1^ PA is defined as any bodily movement involving skeletal muscles that entails energy expenditure, and includes a wide range of activities, such as leisure (walking, dancing, sport) and household tasks (cleaning, gardening). Physical activity plays an important role in the rehabilitation setting, as it can assist with promoting cognitive functioning, emotional well-being and functional independence of patients.^2^ The study by Stolz et al.^2^ highlighted how PA promotes psycho-social benefits, such as self-determination and self-efficacy, in addition to helping with physical recuperation, these being important for reaching rehabilitation objectives, especially those related to going back to work and reintegrating into society.

Speech-language therapists (SLTs) are health care professionals with clinical expertise in managing communication, language, speech, feeding and swallowing-related disabilities, which are frequently connected to neurological disorders that are acquired, including dementia, traumatic brain injury, Parkinson’s disease and stroke.^3^ Occupational therapists (OTs) are health care professionals who focus on improving the functioning of people who have functional limitations.^4^ According to studies conducted in 2022^5^ and 2023,^6^ PA is beneficial for health and function in people who have had a stroke, as it improves motor and cognitive impairments, increases independence in activities of daily living, and reduces the risk of stroke recurrence. Within the context of rehabilitation, there is a noticeable scarcity of empirical studies exploring rehabilitation professionals’ knowledge, attitudes and perceptions towards PA.^6^ The study by Shirley et al.,^7^ based on perspectives from practitioners and students about promoting PA in the physiotherapy setting, highlights that in rehabilitation settings, professionals such as SLTs and OTs often perceive PA as primarily being within the domain of physiotherapy.

While OTs and SLTs have different and distinctive rehabilitation roles, incorporating aspects of PA into both their treatment plans is important, including in neurorehabilitation settings, with recent research highlighting that PA strategies are essential, despite their different professional roles.^8^ In this regard, OTs and SLTs utilise similar clinical reasoning processes when implementing motor learning strategies in adult and paediatric neurorehabilitation, implying that an interdisciplinary focus is appropriate.^9^ Physical activity is not only regarded as important for improving general physical and mental health but also for involvement in daily activities for people of all ages, including those with disabilities.^10^ Occupational therapists use play- and movement-based activities to help children with neurodevelopmental disorders, including autism spectrum disorder (ASD) and attention-deficit/hyperactivity disorder (ADHD), to develop their motor skills, cognitive abilities and social interaction.^11^ Speech-language therapists may incorporate PA through group-based movement exercises that enhance social and linguistic abilities, particularly for children with ASD, or activities that facilitate respiratory control for speech production. However, there is little understanding of how SLTs and OTs view the role of PA in their patient treatment plans. This study, therefore, aimed to explore the use of PA by OTs and SLTs.

## International classification of functioning, disability and health framework

This study uses the World Health Organization’s International Classification of Functioning, Disability and Health (ICF) framework to explore the use of PA by OTs and SLTs in patient treatment. It consists of three components: physical structures and function, activities and participation, and environmental factors. Experts in many fields have adopted it as the accepted paradigm for classifying health conditions, and have used it to direct their choices of assessment instruments and focused interventions, as it offers a thorough method for comprehending disability and health conditions.^8^

The ICF not only focuses on illness and disability, but it also contends that well-being and functioning are important factors that influence health outcomes. A qualitative study conducted in 2022 on the use of the ICF at an inpatient neurorehabilitation facility in the Western Cape, South Africa, indicated that health care professionals in South Africa perceived it to have the potential to improve rehabilitation service delivery due to its focus on guiding and improving interdisciplinary communication.^10^ The ICF provides a holistic focus that links PA to body functions, activity levels, participation, and environmental impacts towards successful integration of PA by OTs and SLTs. This study mainly focused on its three components as follows:

- Body structure and function: this relates to the physiological and anatomical aspects of the body. Impairments in this domain relate to problems in these bodily systems or structures.
- Activity and participation: this relates to an individual’s ability to perform tasks and engage in meaningful life roles. This includes both personal-level activity limitations and broader participation restrictions in societal roles.
- Environmental factors: these factors relate to the physical, social and attitudinal context in which people live. These factors can act as support (facilitators) or obstacles (barriers) to functioning and participation.

## Research methods and design

### Design

The study was conducted using a qualitative approach and a descriptive research design to obtain in-depth information about the participants’ use of PA in patient treatment.^11^ This article builds on a previously published study based on the first author’s thesis entitled *Physical activity in rehabilitation practice: Policy, infrastructure and development perspectives*.

### Recruitment

Convenience sampling was undertaken to recruit OTs and SLTs in the KwaZulu-Natal Province, registered with the Health Professions Council of South Africa and working in the public health sector with either adults or paediatric patients. Recruitment was conducted using designated closed Facebook and WhatsApp groups, which had 3800 and 213 members, respectively, which, to an extent, excluded therapists without access to these platforms. Two invitations were sent on the respective platforms, two months apart.

### Data collection

The participants were interviewed virtually using Zoom and WhatsApp calls to accommodate their availability and ensure accessibility, each taking approximately 45 min to complete. To ensure that the data acquired were adequate to achieve the study’s objective, qualitative data were collected until data saturation was reached.^12^ The interview schedule (Appendix 1) consisted of four sections, the first being their biographical details as context, and the other three relating to the components of the ICF to support a directed content analysis approach.

### Data analysis

Guided by the ICF, data were analysed using a directed content analysis approach. This approach entailed initially using predefined codes based on the ICF framework.^13^ Transcripts were coded using NVivo software, and text segments relating to the predefined categories were coded with a deductive approach:

- Interviews were transcribed verbatim by the first author. The first and second authors read the transcripts three times to ensure a good overall understanding of the content.
- Using the ICF, a coding framework was developed to form the deductive categories.
- Using NVivo software, text segments were coded deductively according to the ICF predefined codes.
- The first and second authors reviewed initial codes and organised them into categories under each ICF component.
- Generated sub-themes were interpreted to explore how PA was understood and applied within each component. Where applicable, verbatim quotes were selected to show therapists’ key concepts and strengthen credibility.
- To ensure scientific rigour, supervision debriefing sessions were held between the first and second authors to confirm interpretations.

### Ethical considerations

The University of KwaZulu-Natal Research Ethics Committee granted ethical approval on 13 August 2024. The ethical clearance number is HSSREC/00007488/2024. Participants were made aware that their participation was voluntary and that they could withdraw at any time during data collection. They gave written consent to participate and for the interviews to be audio-recorded for later transcription and analysis. Participants’ identities were protected, with pseudonyms used and their data kept confidential.

## Results

All those interviewed were employed in the public sector, with 8 of the 10 under the age of 30-years-old. Nine of them were female participants, and only three had more experience than their community service year (Table 1). They worked in both rural and urban districts in KwaZulu-Natal, and all had qualified at South African institutions (Table 1).

**TABLE 1:** Demographic details of participants.

Participant code	Age (years)	Gender	Years’ experience	Profession	District	Urban/Rural
P1	23	F	CS	OT	eThekwini	Urban
P2	23	F	CS	OT	Harry Gwala	Rural
P3	36	F	12	SLT	Ugu	Rural
P4	23	F	CS	OT	eThekwini	Urban
P5	23	M	CS	SLT	eThekwini	Urban
P6	23	F	CS	OT	uMzinyathi	Rural
P7	22	F	CS	SLT	uThukela	Rural
P8	22	F	CS	OT	Harry Gwala	Rural
P9	30	F	6	SLT	eThekwini	Urban
P10	26	F	3	SLT	eThekwini	Urban

OT, occupational therapist; SLT, speech and language therapist; CS, community service; F, female; M, male.

Six sub-themes were generated, two for each of the themes related to the framework, as indicated in Table 2.

**TABLE 2:** Themes and sub-themes.

Themes	Sub-themes	ICF codes	Explanation
1. Body structure and function	Physical endurance and strength building	b7(b730)	Neuro-musculoskeletal and movement-related functions (muscle power)
	Neurological and cognitive enhancement	b1 (b140, b144)	Mental functions (attention, memory)
2. Activities and participation	Engagement in meaningful activities	d4 (d430, d450, d465) d5 (d530, d540, d550, d560) d6 (d630)	Mobility (lifting and carrying objects, walking, moving around using equipment) Self-care (toileting, eating, dressing, drinking) Domestic life (cooking)
	Behavioural and emotional regulation	d7 (730)	Interpersonal interactions and relationships (basic interpersonal reaction)
3. Environmental factors	Resource limitations	e2 (e225)	Natural environment and human-made changes to the environment (climate)
	Interdisciplinary collaboration	e3 (e355, e360)	Support and relationships (health and health-related professionals)

ICF, International Classification of Functioning, Disability and Health.

### Theme 1: Body structure and function

#### Sub-theme 1.1: Physical endurance and strength building

Therapists regarded PA as essential for restoring strength, postural control and respiratory coordination. Many described integrating exercises such as breathing drills and strength training into therapy to address physiological impairments. These were often creatively embedded into routine activities. One therapist shared, ‘We encourage heavy lifting, like groceries for osteoporosis patients because muscle builds bone’ (P1), illustrating how daily tasks were adapted to target functional outcomes. Another added, ‘I assess PA based on the intensity and function […] including the patient’s ability to move, maintain posture for feeding, and complete breathing exercises’ (P10). In summarising the therapeutic role of PA, one participant explained, ‘Physical activity involves muscles used to achieve function through movement’ (P1), reinforcing its integration as a functional intervention rather than a standalone activity.

#### Sub-theme 1.2: Neurological and cognitive enhancement

Beyond physical outcomes, therapists also highlighted the neurological and cognitive benefits of PA. They described how movement-based activities improved memory, attention and sensory regulation, particularly in neurodevelopmental and neurorehabilitation contexts. One therapist noted, ‘Walking increases the size of the hippocampus, aiding memory and learning’ (P3), while another explained, ‘Jumping and spinning provide proprioceptive input to calm ADHD patients’ (P8). The role of PA in supporting cognitive recovery was summarised simply, ‘It also helps improve cognitive function’ (P10). These insights emphasise that PA was not only used to enhance physical recovery but also as a mechanism to stimulate neuroplasticity and promote cognitive engagement in therapy.

### Theme 2: Activities and participation

#### Sub-theme 2.1: Engagement in meaningful activities

Therapists frequently incorporated PA into functional, goal-directed activities to promote engagement and independence in everyday life. These included domestic tasks, sports and daily routines that were meaningful to patients. One participant explained, ‘We use cooking or hanging clothes to integrate range-of-motion exercises into daily life’ (P4), showing how therapy was grounded in real-life function. Motivation was an important factor in promoting participation, as described by one therapist: ‘For a spinal cord injury patient, sport was the only motivator to achieve function’ (P1). Others described how functional PA interventions were adapted to specific clinical needs, ‘A patient with a stroke benefitted from oral motor exercises and posture training, which significantly improved feeding, swallowing safely, and speech intelligibility’ (P10).

Therapists working with children reported adapting PA into structured play or daily routines. One shared, ‘I worked with a child with ASD who struggled with toilet training. We used consistent language during practice, which helped the child become more comfortable and independent at home’ (P9). Similarly, food preparation tasks were used to teach functional skills using household objects: ‘When teaching a child to prepare food, I use familiar, safe household items like plastic plates and child-safe knives’ (P9). These examples show how PA was not just physical exercise – it was purposefully embedded into tasks that promoted skill development, independence and participation.

#### Sub-theme 2.2: Behavioural and emotional regulation

Therapists also used PA to help regulate patients’ behaviour and emotions, particularly when preparing for or transitioning into therapy sessions. Sensory-rich activities such as music and dance were commonly used to create a calm and responsive environment, especially for children. As one therapist shared, ‘Music and dancing help calm children with autism before sessions’ (P3). Another added, ‘Physical activity improves mood and social skills for psychiatric patients’ (P8), highlighting its emotional and interpersonal benefits.

Establishing therapeutic rapport and emotional readiness was seen as a prerequisite for effective participation, especially in paediatric therapy. One therapist explained, ‘It usually takes multiple sessions to build a relationship with the child before we can begin therapy activities. I always make sure to involve the parents in this process’ (P9). These reflections underscore the dual role of PA in both therapeutic function and emotional engagement.

### Theme 3: Environmental factors

#### Sub-theme 3.1: Resource limitations

Participants described several challenges related to space, equipment and infrastructure that limited their ability to deliver PA interventions effectively. Creative solutions were often necessary to work around these constraints. ‘We use recyclable materials, like paper, to create therapy balls when resources are scarce’ (P8), shared one therapist. Others noted the impact of weather and shared facilities: ‘Shared therapy spaces and bad weather restrict activities’ (P8), and ‘Environments to work in, especially in wards… it would have been nice to have isolated therapy rooms’ (P10). These examples reflect how systemic limitations frequently restricted the type and consistency of PA that could be delivered in public-sector settings.

#### Sub-theme 3.2: Interdisciplinary collaboration

Despite these challenges, therapists emphasised the importance of interdisciplinary teamwork and caregiver support in enabling effective PA integration. ‘Joint sessions with physiotherapists clarify overlapping roles’ (P1), one participant explained, highlighting the value of collaborative treatment planning. Another added, ‘Educating caregivers ensures patients comply with physical activity at home’ (P4). However, this collaboration was not always present, with one participant stating, ‘Working as an interdisciplinary team in session is lacking.’

Therapists also recommended strategies to extend PA beyond the clinic. One suggested, ‘Home visits make it easier for the child to do physical activities […] being outside, in familiar spaces, improves the child’s skills’ (P9), advocating for therapy approaches that support environmental continuity. These insights suggest that supporting PA in rehabilitation requires systemic flexibility, cross-disciplinary communication, and community-based implementation.

## Discussion

Regarding theme 1, sub-theme 1, pertaining to the development of physical endurance and strength, a participant noted that they address deficiencies in body structure through the utilisation of specific physical activities, such as endurance exercises, strength training, or weightlifting, to enhance bone density, muscle strength, and motor function. This aligns with the study conducted by Benedetti and colleagues in 2018, who recommended weightlifting and progressive resistance training to improve muscle strength. This research aligns with a previous study,^14^ which emphasises the importance of incorporating functional application of PA into daily chores during rehabilitation. While previous research has focused on physiological workout characteristics, our findings highlight the importance of incorporating these exercises into daily chores (e.g. feeding), which improves both clinical outcomes and real-world application.^15^

Regarding theme 1, sub-theme 2, physical endurance and strength building, the participants felt that PAs, such as walking, can improve cerebral functioning, particularly in domains such as memory and learning.^16^ One participant noted that walking helps with memory and learning, as moderate exercise can expand the hippocampus, which is essential for memory formation and cognitive function, as supported by findings from the existing literature.^17^ Physical activity promotes sensory integration, relaxation for patients with ADHD,^18^ and enhances overall brain functioning by improving proprioception, which is essential for motor control and coordination.^17^

Three participants spoke about how engaging in meaningful activities, such as cooking, gardening and athletics, is essential for helping rehabilitation patients regain their functional independence and participate in daily life. Research supports this strategy by showing that incorporating fun, useful tasks into therapy can improve physical capability, motivation and general well-being.^18^ Sports can be a strong motivation for functional recovery, and integrating range-of-motion exercises into routine activities, such as cooking or housework, shows a practical, goal-oriented approach to therapy. Two participants in this study highlighted the value of establishing a supportive atmosphere that promotes involvement. This is consistent with research demonstrating that activity-based therapies promote improved independence, cognitive function and quality of life, particularly when customised to the requirements and interests of the individual.^19^ Overall, our results support therapeutic outcomes and patient participation in daily life by demonstrating the need for meaningful, real-world activities in effective rehabilitation.

According to three participants, using sensory and PA can assist patients to prepare for therapy and social interaction by supporting behavioural and emotional regulation. For instance, dancing and music are used to help autistic youngsters relax before sessions, while exercise helps psychiatric patients feel better and interact with others. These results are consistent with studies that highlight the advantages of multimodal stimulation for neuropsychiatric conditions.^20^ In older people with neurocognitive problems, multisensory stimulation reduces agitation and anxiety, with mood gains being less consistent.^20^ Dance-based therapies have been demonstrated to improve motor, cognitive and social functioning.^21^ Additionally, multisensory integration promotes cognitive and emotional processing, which makes learning and emotional regulation easier.^20^ Multisensory treatment has been acknowledged for its effectiveness in psychiatric care and is known to foster positive social and emotional results.^21^ These results highlight how important it is to include sensory experiences in therapy to improve patient outcomes.

Regarding theme 3, sub-theme 1, resource limitations, this was reported to include a lack of equipment and space, which required the therapists to be creative, but frequently restricted the range of physical activities that could be undertaken. One participant stated that when resources are limited and activities are restricted by shared therapy facilities and bad weather, they make therapeutic balls out of paper and play indoors. A recent study supports these findings by emphasising how environmental limitations affect medical procedures, and how hospital settings affect patients’ levels of PA.^22^ Researchers have noted that addressing environmental factors in rehabilitation is essential for sustainable health care practices, highlighting the need to use creative methods to overcome resource constraints.^23^ The need for appropriate therapeutic environments was a recurring theme among participants, as highlighted by those who stated that environments to work in, especially wards, should ideally have isolated therapy rooms to work on. The use of temporary solutions may restrict the range and efficacy of PA and, therefore, impair patient participation and results. This emphasises the need for access to adequate facilities and resources to enable therapists to engage in a variety of physical activities. Addressing resource constraints is essential for improving the quality of rehabilitation services and ensuring that patients receive thorough and effective treatment.

Regarding theme 3, sub-theme 2, interdisciplinary collaboration among professionals and caregivers, the participants felt that this was essential to develop a supportive atmosphere that promotes the incorporation of physical activities into therapy. They felt that collaborative sessions with physiotherapists ensured a unified approach to treatment by addressing overlapping areas of responsibility. Participants also mentioned that to promote patient compliance with PA at home, caregiver education is essential. These findings have been reinforced by a recent study that emphasises the value of caregiver participation in encouraging physical exercise in older persons with Alzheimer’s disease.^24^ A study highlights that educating caregivers about physical therapy programmes increases their capacity to assist patients in an efficient manner, which results in improved outcomes and greater adherence to recommended activities.^25^ Maximising the incorporation of PA into therapeutic procedures requires cooperation between caregivers and health care professionals,^26^ with this study finding that introducing home visits into treatment methods can improve interdisciplinary teamwork. One participant mentioned that home visits enable therapists to interact with children in their natural circumstances, making therapy more relevant and practical by addressing real-life issues and encouraging PA in familiar settings. Empowering families can assist with the successful implementation of PA and long-lasting therapeutic solutions, as involving parents and caregivers in therapy can strengthen the collaborative partnership with therapists. Encouraging cooperation between therapists and families can make it possible for parents or other caregivers to take an active role in therapy and carry out PA more successfully.

## Limitations

The following limitations have been identified:

- Most participants were community service therapists with limited clinical experience, which may have impacted the depth of their perspectives.
- Recruitment was limited to Facebook and WhatsApp groups, excluding therapists without access to these platforms and introducing possible selection bias.
- Data were collected from a limited number of districts in KwaZulu-Natal, so findings may not be generalisable to other regions or provinces.

## Conclusion

This study demonstrates the contribution of PA as a flexible and effective rehabilitation strategy for SLTs and OTs. Grounded in the ICF framework, it shows the importance of integrating PA to enhance emotional, cognitive and functional outcomes. Therapists creatively adapted PA interventions despite limited resources, highlighting the need for accessible, context-relevant tools in public health care settings. Investment in infrastructure, funding and resource availability is essential to support this integration. The findings also emphasise the importance of interdisciplinary collaboration and caregiver involvement in reinforcing treatment beyond clinical settings. By addressing individual functional needs and environmental barriers, therapists can deliver more patient-centred and goal-oriented care. Adopting an ICF-informed, team-based approach to PA in rehabilitation can strengthen service delivery, promote patient engagement, and improve health outcomes in resource-constrained contexts.

## Appendix 1

### Semi-structured interview schedule

#### Body structure and function

- Understanding PA: What is your understanding of PA in the context of rehabilitation? Can you provide examples of physical activities that you find relevant in your clinical practice?
- Classification and application in clinical practice: How do you assess and classify the levels of PA in your patients during treatment sessions? Could you share specific examples of how you monitor PA levels in your clinical practice?

### Activity and participation

- Role of PA in rehabilitation: What is the role of PA in the treatment process for your patients? Can you share a patient case or experience where PA played a crucial role in their rehabilitation outcome?
- Integration of PA into therapy: How do you integrate PA into your daily treatment sessions? Can you elaborate on specific strategies or techniques you use to encourage PA during rehabilitation?

### Environmental factors

- Tools and resources: What tools, equipment or resources do you use to promote PA in your treatment sessions? Are there any specific guidelines or frameworks you follow to ensure PA is appropriately incorporated intoyour therapy?
- Barriers and challenges (environmental factors): What challenges do you face when trying to incorporate PA into your clinical practice? How do you overcome them?
- Closing remarks: Is there anything you would like to share about the role of PA in your practice? Do you have any suggestions or recommendations for improving the integration of PA into rehabilitation?
